# Nomograms that predict the survival of patients with adenocarcinoma in villous adenoma of the colorectum: a SEER-based study

**DOI:** 10.1186/s12885-020-07099-3

**Published:** 2020-06-29

**Authors:** Chao-Tao Tang, Ling Zeng, Jing Yang, Chunyan Zeng, Youxiang Chen

**Affiliations:** Department of Gastroenterology, the First Affiliated Hospital of Nanchang University, 17 Yongwaizheng Street, Nanchang, 330006 Jiangxi China

**Keywords:** Adenocarcinoma in villous adenoma, Colorectum, Nomogram, Survival, SEER

## Abstract

**Background:**

Considering that the knowledge of adenocarcinoma in villous adenoma of the colorectum is limited to several case reports, we designed a study to investigate independent prognostic factors and developed nomograms for predicting the survival of patients.

**Methods:**

Univariate and multivariate Cox regression analyses were used to evaluate prognostic factors. A nomogram predicting cancer-specific survival (CSS) was performed; internally and externally validated; evaluated by receiver operating characteristic (ROC) curve, C-index, and decision curve analyses; and compared to the 7th TNM stage.

**Results:**

Patients with adenocarcinoma in villous adenoma of the colorectum had a 1-year overall survival (OS) rate of 88.3% (95% CI: 87.1–89.5%), a 3-year OS rate of 75.1% (95% CI: 73.3–77%) and a 5-year OS rate of 64.5% (95% CI: 62–67.1%). Nomograms for 1-, 3- and 5-year CSS predictions were constructed and performed better with a higher C-index than the 7th TNM staging (internal: 0.716 vs 0.663; *P* < 0.001; external: 0.713 vs 0.647; *P* < 0.001). Additionally, the nomogram showed good agreement between internal and external validation. According to DCA analysis, compared to the 7th TNM stage, the nomogram showed a greater benefit across the period of follow-up regardless of the internal cohort or external cohort.

**Conclusion:**

Age, race, T stage, pathologic grade, N stage, tumor size and M stage were prognostic factors for both OS and CSS. The constructed nomograms were more effective and accurate for predicting the 1-, 3- and 5-year CSS of patients with adenocarcinoma in villous adenoma than 7th TNM staging.

## Background

According to global cancer statistics in 2018, colorectal cancer (CRC) is the third most common cancer, with 97,220 new cases of colon cancer and 43,030 new cases of rectal cancer worldwide [1]. There are three pathways involved in the pathogenesis of sporadic CRC: the classic colorectal adenoma (CRA)-adenocarcinoma pathway, the de novo pathway and the inflammatory cancer pathway. Among these pathways, the adenoma-adenocarcinoma pathway is the most common mechanism for the development of CRC [2]. Adenomatous polyps account for approximately 60–70% of all colonic polyps and are divided into tubular adenomas, villous/tubulovillous adenomas (VA/TVAs), sessile serrated adenomas (SSAs) and traditional serrated adenomas (TSAs), while TSAs are often admixed with SSA and VA/TVA [3]. The pathological characteristic of villous adenoma is more than 75% of villous features with or without epithelial projections. According to previous studies, compared with other adenomas, adenomas with villous features have been considered a risk factor associated with an increased probability of developing into a more advanced neoplasia or dysplasia lesion [4]. Moreover, the size of the adenoma and the number of adenomas increase the risk of advanced development [5]. The results of a multicenter cohort study suggested that adenomas of more than 2 cm in diameter and with high-grade dysplasia were highly correlated with the development of CRC (HR: 9.25, 95% CI, 6.39–13.39) [6]. Although mounting evidence has suggested that villous adenoma is correlated with adenocarcinoma, current knowledge of the survival rate of patients with adenocarcinoma in villous adenoma is limited to a small series of studies [7–11]. The first report was that a 19-year-old male had carcinoma arising from a villous adenoma [12]. According to a recent case report, a 71-year-old female patient with intramucosal adenocarcinoma in villous adenoma recurred after 19 months in the ulcer scar site because of the careless pathological examination. After post-endoscopic submucosal dissection (ESD), there were no recurrent signs during 9 years of follow-up [10]. Hence, identifying prognostic factors for patients with adenocarcinoma in villous adenoma is a vital part of the assessment and therapy of CRC.

The Surveillance, Epidemiology, and End Results (SEER) program contains detailed research data on many kinds of tumors that cover almost 30% of the population in the United States [13]. Additionally, nomograms are widely used to assess the prognosis of cancers because of their ability to transform a statistical predictive model into a single numerical estimate of the probability of an event, which is a user-friendly method that guides clinical decision-making for doctors [14]. Therefore, in our study, we utilized a nomogram to analyze the impact of clinical characteristics such as TNM stage and tumor size on the survival rate of patients with adenocarcinoma in villous adenoma using the SEER database.

## Methods

### Data source

A total of 970,163 patients with CRC were identified from 2004 to 2015. All data were extracted from the SEER database of the United States, which covers abundant information on cancers. SEER^*^Stat software (version 8.3.6, downloaded from http://seer.cancer.gov/seerstat/) was used to extract patient information from the SEER database.

### Population selection

**Fig. 1:**
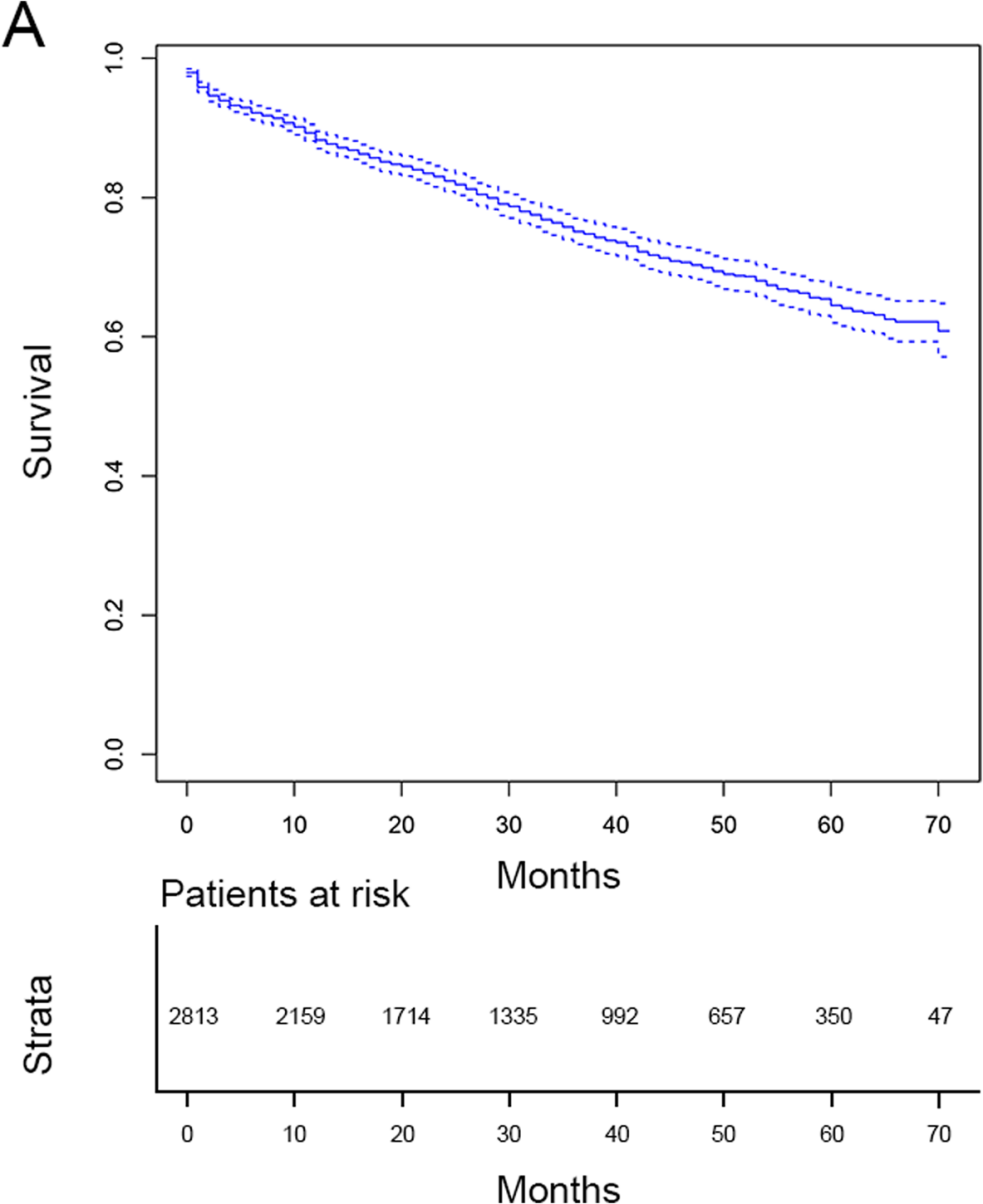
OS curves for the patients

To acquire the necessary information from the databanks, we established criteria to exclude some useless data. As shown in Fig. 1, we carefully reviewed the patient information. The inclusion criteria were as follows: (1) positive pathological diagnosis; (2) sufficient information about survival; and (3) available follow-up data. The exclusion criteria were as follows: (1) pathological diagnosis not adenocarcinoma in villous adenoma (ICD-O-3 Hist/behav, malignant: 8261/3); (2) no detailed information about the specific cause of death or other cause of death; (3) no information on AJCC TNM status; (4) unknown race of patient; and (5) no record of tumor number and pathological grade. The missing value were listed in the Supplementary Table 1.

### Study variables

Several variables were extracted from the SEER database, including age, race, sex, T stage, N stage, M stage, pathological grade of the tumor, number of tumors and tumor size. Patients were divided by age into < 50 years, 50–59 years, 60–69 years and > =70 years. Race was classified as black, white, and other. Pathological grade was categorized as well differentiated (grade I), moderately differentiated (grade II), poorly differentiated (grade III), and undifferentiated (anaplastic, grade IV). The T stage was divided into Tis, T1, T2, T3, T4 and TX. The N stage was described as N0 (No), N1 (Yes), N2 (Yes) and NX. For M stage, M0 indicated negative metastasis, while M1 indicated positive metastasis. Tumor size was separated into < 5 cm, > = 5 cm and unknown. The number of tumors was divided into two groups: 1 tumor or more than 1 tumor.

### Statistical analysis

As described in the previous section, the demographic characteristics and clinicopathological information of the patients are summarized in Table 1. Differences in the baseline characteristics between patients who died from cancer and patients who died from other causes were assessed by the chi-square test. Overall survival (OS) and cancer-specific survival (CSS) were regarded as the primary indexes of our study. The potential factors associated with OS and CSS were analyzed by univariate and multivariate Cox regression analyses. Survival curves were obtained by the K-M method and stratified by the clinicopathological index. To perform the nomogram, first, we performed the multivariate Cox regression analysis by the “coxph” function in the “survival” package; after that, we performed the “step” function to determine the value of the Akaike Information Criterion (AIC), which is a well-known method for selecting variables; according to the AIC value, we determined the variables to build the nomogram; finally, we used the “plot” function and “nom” function in the “rms” packages to construct the nomogram model. The survival curves, ROC curves, C-index and calibration curves were calculated using the “rms”, “foreign” and “survival” packages in R software (Version 3.5.0). A competing-risk model was established via the “cmprsk” package. All packages used in our manuscript were obtained from the website (https://www.r-project.org/) All results were considered to be statistically significant when the *P* value was less than 0.05.

**Table 1:** Patients’ demographics, clinical characteristics at diagnosis

Variables	Total (%)	Cause-specific Death (%)	Death due to other causes (%)	*P* Value
n	2813	398	268	
Age				< 0.0001
< 50	309 (10.98%)	32 (8.04%)	4 (1.49%)	
50–59	643 (22.86%)	64 (16.08%)	24 (8.96%)	
60–69	711 (25.28%)	96 (24.12%)	39 (14.55%)	
≥ 70	1150 (40.88%)	206 (51.76%)	201 (75%)	
Race				0.375
White	2265 (80.52%)	319 (80.15%)	222 (82.84%)	
Black	318 (11.3%)	59 (14.82%)	30 (11.19%)	
Other	230 (8.18%)	20 (5.03%)	16 (5.97%)	
Sex				0.8802
Male	1466 (52.12%)	210 (52.76%)	143 (53.36%)	
Female	1347 (47.88%)	188 (47.24%)	125 (46.64%)	
Pathology Grade				0.013
I	492 (17.49%)	49 (12.31%)	38 (14.18%)	
II	2037 (72.41%)	273 (68.59%)	203 (75.75%)	
III	220 (7.82%)	58 (14.57%)	23 (8.58%)	
IV	64 (2.28%)	18 (4.52%)	4 (1.49%)	
Lymph node metastasis				< 0.0001
NO	1993 (70.85%)	195 (48.99%)	202 (75.37%)	
Yes	758 (26.95%)	181 (45.48%)	55 (20.52%)	
NX	62 (2.2%)	22 (5.53%)	11 (4.1%)	
Metastasis				< 0.0001
No	2559 (90.97%)	242 (60.8%)	251 (93.66%)	
Yes	254 (9.03%)	156 (39.2%)	17 (6.34%)	
Tumor size				< 0.0001
≤ 5 cm	1596 (56.74%)	156 (39.2%)	148 (55.22%)	
> 5 cm	680 (24.17%)	157 (39.45%)	57 (21.27%)	
Unknow	537 (19.09%)	85 (21.36%)	63 (23.51%)	
Tumor number				0.11
1	2557 (90.9%)	350 (87.94%)	224 (83.58%)	
> 1	256 (9.1%)	48 (12.06%)	44 (16.42%)	
T stage				< 0.0001
Tis	146 (5.19%)	3 (0.75%)	11 (4.10%)	
T1	904 (32.14%)	58 (14.57%)	92 (34.33%)	
T2	521 (18.52%)	53 (13.32%)	56 (20.90%)	
T3	921 (32.74%)	159 (39.95%)	85 (31.72)	
T4	244 (22.86%)	91 (22.86%)	9 (3.36%)	
Tx	77 (2.74%)	34 (8.54%)	15 (5.6%)	

## Results

### Patient characteristics

As depicted in Supplementary Figure 1, according to the criteria set at the beginning of our study, we finally extracted 2813 patients who were diagnosed with adenocarcinoma in villous adenoma by histopathology from the SEER database. Table 1 lists the basic information regarding the demographic and clinical characteristics of the patients with adenocarcinoma in villous adenoma. As shown in Table 1, of the 2813 patients, 666 died from different causes, including carcinoma and other causes. Among these patients, 398 patients died from adenocarcinoma, and 268 patients died due to other causes. In the whole cohort, the six variables of age, grade, tumor size, T stage, N stage and metastasis had statistical significance in the cases of death attributed to adenocarcinoma and other causes, while no significant differences were observed for race, sex or tumor number.

### Survival analysis

As shown in Fig. 1 and Table 2, overall, the patients had a 1-year OS of 88.3% (95% CI: 87.1–89.5%), 3-year OS of 75.1% (95% CI: 73.3–77%) and 5-year OS of 64.5% (95% CI: 62–67.1%). As shown in Table 2, some characteristics, such as age, TNM stage and pathological grade, suggested that advanced tumors highly affected survival, while we also found that the size and number of tumors had an effect on the prognosis of patients. The larger the tumor and the greater the number of tumors, the shorter the survival time is. In line with the results shown in Table 2, the analysis of OS by Kaplan-Meier plots revealed that age, race, pathological grade, N stage, T stage, metastasis, tumor size and tumor number were prognostic factors (Supplementary Figures 2, 3 and 4). Subsequently, we performed univariate and multivariate Cox regression analyses for OS and CSS (Tables 3 and 4). With regard to OS, in multivariate analysis, age, race, T stage, metastasis, tumor size and tumor number were identified as prognostic factors. For example, compared to patients more than 70 years old, patients who were less than 50 years old were obviously associated with a lower mortality risk (HR: 0.175, 95% CI: 0.123–0.249). Black race, advanced T stage and M stage, larger tumor number and tumor size were also hazardous factors for survival. For CSS, multivariate analyses revealed that some variables, including age, race, T stage, pathological grade, N stage, tumor size and metastasis, remained prognostic factors. Furthermore, based on the competing-risk model, the CSS curves showed that age, race, T stage, pathological grade, N stage, tumor size and M stage were potential prognostic factors (Supplementary Figures 5, 6 and 7).

**Table 2:** 1-, 3- and 5-year survival of OS among patients according to different hierarchical analysis

Variables	1-year (%) (95% CI)	3-year (%) (95% CI)	5-year (%) (95% CI)	log-rank test
All patients	88.3%(87.1–89.5%)	75.1%(73.3–77%)	64.5% (62–67.1%)	–
Age				*P* < 0.0001
< 50	96.2% (94–98.6%)	86.3% (81.8–91.1%)	80.8% (74.6–87.5%)	
50–59	93.5% (91.5–95.5%)	85.6% (82.4–88.9%)	77.5% (72.9–82.4%)	
60–69	92.8% (90.9–94.8%)	78.4% (4.8–82.2%)	71.5% (67–76.2%)	
≥ 70	80.4% (78.1–82.9%)	64.4% (61.3–67.6%)	48.6% (44.5–53.6%)	
Race				*P* = 0.02
White	88.4% (87–89.8%)	75% (73–77.1%)	63.9% (61–66.8%)	
Black	86% (82.2–90%)	71.4% (66–77.3%)	60.8% (54–68.6%)	
Other	90.8% (86.8–94.8%)	81.7% (75.9–88.1%)	74.2% (65.1–84.5%)	
Sex				*P* = 0.3
Male	88.6% (87–90.4%)	73.8% (71.2–76.5%)	63.1% (59.6–66.9%	
Female	87.9% (86.1–89.7%)	76.4%(73.9–79.1%)	65.9%(62.3–69.6%)	
Pathology Grade				*P* < 0.0001
I	90.6% (88–93.4%)	82.4%(78.6–86.4%)	72.1%(66.3–78.5%)	
II	89.3% (88.7–91.4%)	75.2%(73.9–78.2%)	64.1%(61.2–67.3%)	
III	77.5% (72.1–83.4%)	62.1%(54.9–68.5%)	53.4%(43.9–62.4%)	
IV	77.5%(67.4–89.2%)	58.1%(45.5–74.5%)	–	
N Stage				*P* < 0.0001
No	90.8%(89.5–92.2%)	79.5% (77.4–81.6%)	69% (66.1–72.1%)	
Yes	80.7% (75.3–86.5%)	52.6% (37.6–55%)	31.3% (22.4–43.7%)	
Unknown	60.7% (47.2–73.1%)	43.9 (32.24%-59,8%)	35.1% (20.5–60.1%)	
Metastasis				*P* < 0.0001
No	91.3% (90.1–92.4%)	80.4% (78.7–82.3%)	69.7% (67.1–72.4%)	
Yes	58.7% (52.7–65.4%)	22.5% (17.2–29.5%)	13.3% (8.15–21.6%)	
Tumor size				
≤ 5 cm	91.6% (90.1–93.1%)	81.6% (78.8–83.6%)	70.7% (67.2–74.4%)	*P* < 0.0001
> 5 cm	85.1% (82.5–87.7%)	65% (61.1–69%)	53.8% (49–58.9%)	
Unknow	84.3% (81.1–87.5%)	72.5% (67–75.7%)	62.4% (57.2–68.1%)	
Tumor number				*P* = 0.004
1	88.3% (87–89.6%)	76.2% (74.2–78.1%)	65.5% (62.8–68.3%)	
> 1	88.7% (84.8–92.7%)	67.2% (61.3–73.8%)	56.5% (49.6–64.4%)	
T stage				
Tis	91% (87.3–96.6%)	79.9% (72.5–88.1%)	–	*P* < 0.0001
T1	89.2%(87.1–91.3%)	78.5% (75.5–81.7%)	65.8% (61.3–70.7%)	
T2	88.1% (85.3–91.1%)	75.7% (71.6–80%)	64.3% (59.1–71.1%)	
T3	89.6% (87.6–91.7%)	75% (72.1–78.7%)	66% (61.9–70.5%)	
T4	82.1% (77.2–87.3%)	62.5%(55.6–70.3%)	55.1% (46.5–65.3%)	
Tx	75.4% (65.8–86.4%)	56% (44.9–72.6%)	–

**Table 3:** Univariate analysis and Multivariate analysis of variables for OS in patients

Variables	Univariate analysis		Multivariate Analysis	
	HR (95%CI)	*P* value	HR (95%CI)	*P* value
Age				
< 50	0.282(0.201–0.397)	0.000	0.175(0.123–0.249)	0.000
50–59	0.335(0.266–0.422)	0.000	0.281(0.222–0.355)	0.000
60–69	0.48(0.395–0.583)	0.000	0.376(0.307–0.459)	0.000
≥ 70	Reference	–	Reference	–
Race				
Other	0.585(0.397–0.861)	0.007	0.524(0.355–0.774)	0.001
White	0.865(0.691–1.083)	0.205	0.794(0.633–0.995)	0.045
Black	Reference	–	Reference	–
Sex				
Male	1.08(0.927–1.257)	0..323	–	–
Female	Reference	–	–	–
Pathology Grade				
I	0.416(0.261–0.664)	0.000	0.758(0.47–1.223)	0.256
II	0.573(0.374–0.880)	0.011	0.943(0.61–1.456)	0.789
III	0.980(0.612–1.57)	0.932	1.428(0.887–2.3)	0.142
IV	Reference	–	Reference	–
N stage				
No	0.7(0.579–0.846)	0.000	0.887(0.717–1.072)	0.199
Yes	Reference	–	Reference	–
Unknown	2.0(1.574–2.543)	0.000	1.688(1.305–2.133)	0.000
Metastasis				
No	0.161(0.135–0.192)	0.000	0.17(0.138–0.208)	0.000
Yes	Reference	–	Reference	–
Tumor size		0.000		0.000
≤ 5 cm	0.518(0.436–0.615)	0.000	0.731(0.608–0.879)	0.001
> 5 cm	Reference	–	Reference	–
Unknow	0.787(0.643–0.964)	0.021	1.081(0.872–1.338)	0.478
Tumor number				
1	0.725(0.582–0.904)	0.004	0.76(0.609–0.950)	0.016
> 1	Reference	–	Reference	–
T stage				
Tis	0.511(0.332–0.787)	0.002	0.624(0.402–0.968)	0.035
T1	0.573(0.441–0.746)	0.000	0.782(0.596–1.028)	0.078
T2	0.642(0.484–0.853)	0.002	0.867(0.648–1.160)	0.336
T3	0.622(0.48–0.807	0.000	0.687(0.528–0.894)	0.005
T4	Reference	–	Reference	–
Tx	1.239(0.805–1.908)	0.331	1.442(0.929–2.238)	0.102

**Table 4:** Univariate analysis and Multivariate analysis of variables for CSS in patients

Variables	Univariate analysis		Multivariate Analysis	
	HR (95%CI)	*P* value	HR (95%CI)	*P* value
Age		0.000		0.000
< 50	0.5(0.344–0.725)	0.000	0.238(0.161–0.352)	0.000
50–59	0.486(0.367–0.643)	0.000	0.373(0.281–0.496)	
60–69	0.679(0.533–0.866)	0.002	0.468(0.363–0.602)	
≥ 70	Reference	–	Reference	–
Race		0.019		0.024
Other	0.492(0.296–0.817)	0.006	0.509(0.305–0.849)	0.01
White	0.77(0.583–1.017)	0.066	0.754(0.569–0.998)	0.049
Black	Reference	–	Reference	–
Sex		0.535	–	–
Male	1.064(0.874–1.296)	0.535	–	–
Female	Reference	–		
Pathology Grade		0.000		0.001
I	0.291(0.17–0.50)	0.000	0.665(0.381–1.159)	0.15
II	0.406(0.252–0.655)	0.000	0.786(0.483–1.28)	0.333
III	0.867(0.511–1.471)	0.596	1.348(0.788–2.308)	0.276
IV	Reference	–	Reference	–
Lymph node		0.000		
No	0.468(0.369–0.592)	0.000	0.691(0.538–0.888)	0.004
Yes	Reference	–	Reference	–
Unknown	2.074(1.574–2.733)	0.000	1.577(1.186–2.098)	0.002
Metastasis				
No	0.089(0.072–0.109)	0.000	0.114(0.089–0.146)	0.000
Yes	Reference	–	Reference	–
Tumor size		0.000		0.000
≤ 5 cm	0.365(0.292–0.457)	0.000	0.618(0.486–0.786)	0.000
> 5 cm	Reference	–	Reference	–
Unknow	0.642(0.496–0.831)	0.001	0.993(0.755–1.306)	0.96
Tumor number				
1	0.841(0.622–1.138)	0.262	–	–
> 1	Reference	–	–	–
T stage		0.000		0.000
Tis	0.28(0.151–0.519)	0.000	0.435(0.232–0.817)	0.01
T1	0.406(0.297–0.555)	0.000	0.702(0.505–0.976)	0.035
T2	0.459(0.326–0.646)	0.000	0.773(0.542–1.104)	0.157
T3	0.478(0.595–1.701)	0.000	0.56(0.41–0.763)	0.000
T4	Reference	–	Reference	–
Tx	1.006(0.595–1.701)	0.981	1.248(0.728–2.139)	0.421

### Performance of the nomograms

**Fig. 2:**
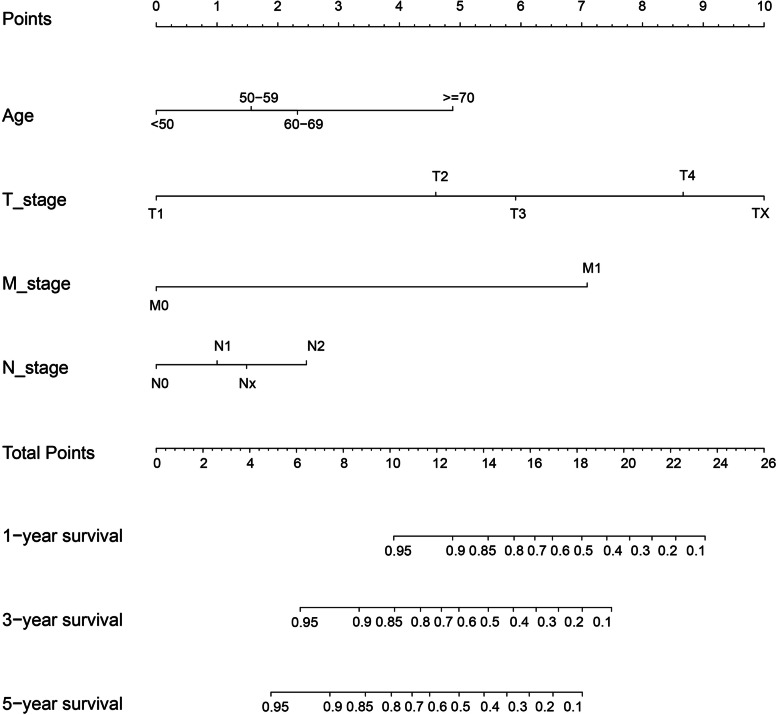
A nomogram for the prediction of the 1-, 3- and 5-year OS rates of patients with adenocarcinoma in villous adenoma

To construct a survival prediction model, we selected CSS as the main observation and then built a nomogram plot. As listed in Table 4, patients with age > 70 years, advanced T stage, distant metastasis, positive LNM and larger tumor size (> 5 cm) and black patients had worse prognosis. To build the nomogram, race and tumor size were not included because the AIC value was obviously larger when it was added into the nomogram. Therefore, we established a nomogram based on four other prognostic factors (Fig. 2). According to the nomogram, we found that T stage contributed the most to the prognosis of AC patients, followed by M stage and age, whereas positive LNM had the least proportion for predicting survival. To explain the nomogram, a straight line can be drawn down to each time point to determine the estimated probability of survival. With respect to each predictor, we could read the points assigned on the 0–10 scale at the top and then add these points. The corresponding predictions of 1-, 3-, and 5-year risk are read last by finding the number on the “Total Points” scale.

### Validation of the nomogram model

**Fig. 3:**
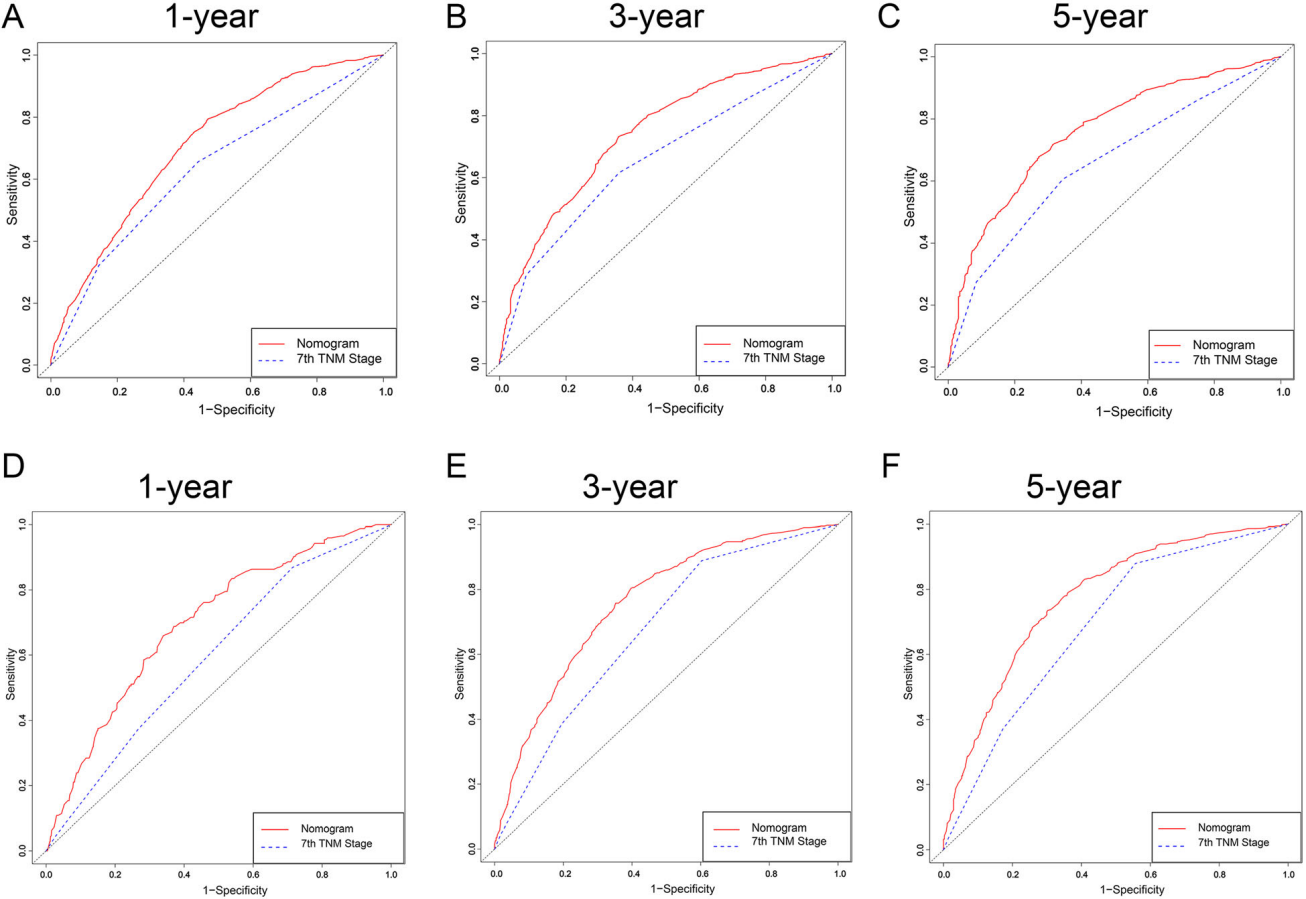
ROC curve of the nomogram and 7th TNM stage in predicting the prognosis of patients from 2004 to 2015. **a-c** ROC curve for the 1-, 3- and 5-year points in the 2004–2009 cohort. **d-f** ROC curve for the 1-, 3- and 5-year points in the 2010–2015 cohort

**Fig. 4:**
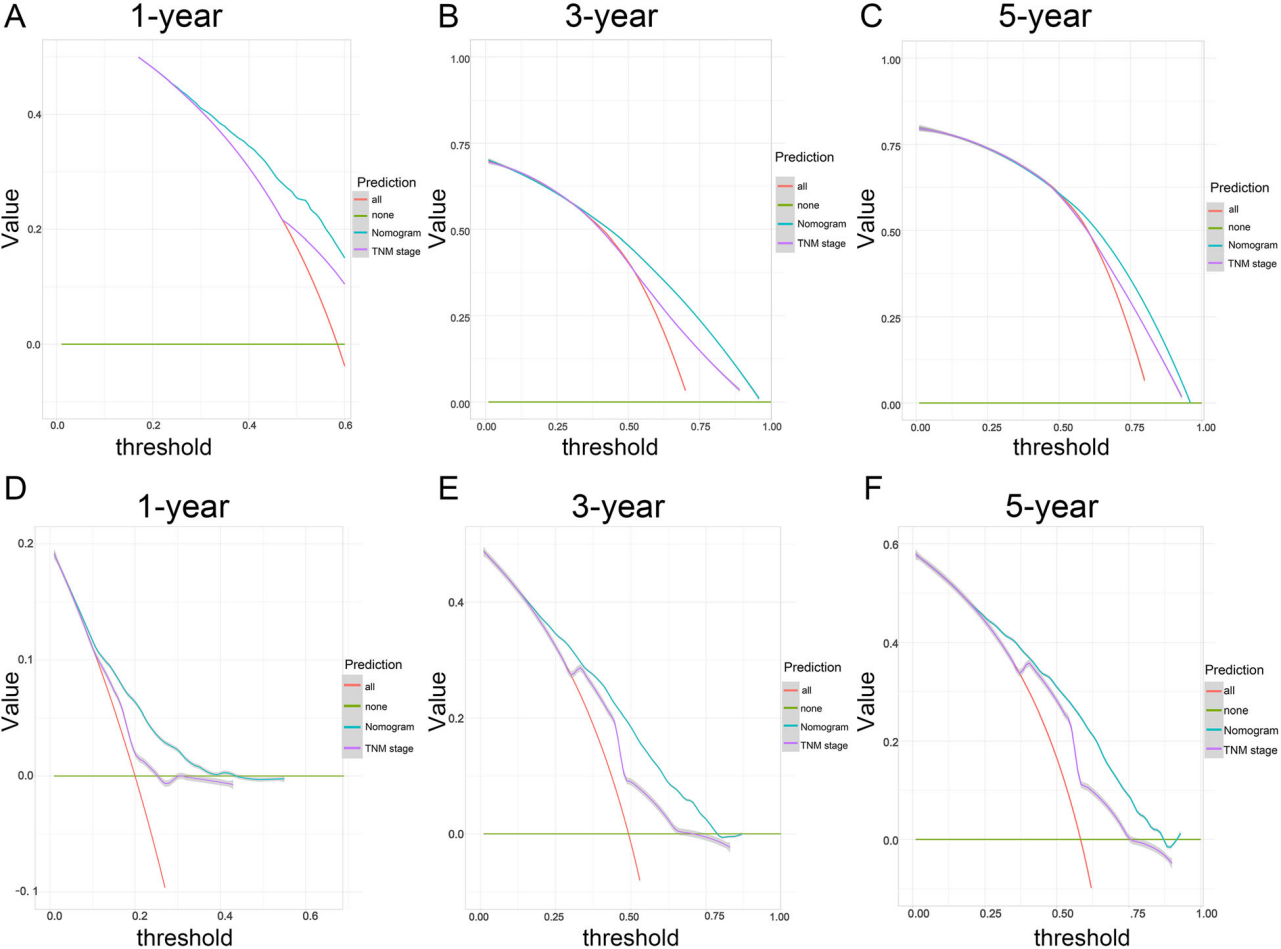
Decision curve analysis for the nomogram and the 7th TNM stage model in the prediction of patient prognosis. **a-c** 1-, 3- and 5-year points in the 2004–2009 cohort. **d-f** 1-, 3- and 5-year points in the 2010–2015 cohort

To investigate the validity of the nomogram, we divided the patients into internal and external cohorts according to the year of diagnosis (2004–2009 group and 2010–2015 group) and determined the C-index value. As listed in Table 5, the value of the C-index in the internal cohort was 0.716 (95% CI, 0.684–0.773), which was higher than the TNM stage value (C-index, 0.663, 95% CI, 0.603–0.734), suggesting that the nomogram was more effective for predicting survival than TNM stage. In line with the results of the external cohort, the nomogram was superior to TNM stage (external cohort, 0.713, 95% CI, 0.641–0.794; TNM stage, 0.647, 95% CI, 0.611–0.709). With respect to the specificity and sensitivity of the nomogram, in the internal cohort, we found that the AUC values for predicting 1-year, 3-year and 5-year survival by the nomogram were 0.701 (0.612–0.751), 0.771 (0.672–0.811) and 0.762 (0.673–0.821), respectively, while the TNM stage values for predicting 1-year, 3-year and 5-year survival were 0.596 (0.537–0.702), 0.683 (0.601–0.724) and 0.689 (0.634–0.758), respectively (Table 5). Compared to the TNM stage model, the nomogram was better at predicting prognosis at 1 year, 3 years and 5 years (Fig. 3a-c). As indicated by the external cohort, the nomogram also performed better than TNM stage (1-year AUC: 0.689 vs. 0.643, 3-year AUC: 0.764 vs. 0.714, 5-year AUC: 0.771 vs. 0.703, *P* < 0.001, Table 5 and Fig. 3d-f). Furthermore, to compare the clinical usability between the nomogram and TNM stage, we performed a DCA plot. As shown in Fig. 4, in both the internal cohort and the external cohort, the predictive efficiency of the nomogram was better than that of TNM stage for 1-year, 3-year and 5-year survival.

**Table 5:** Accuracy of the prediction score of the nomogram and TNM stage for estimating prognosis of patients

Variable	Value (95%CI)	
	Internal validation	External validation
C index for nomogram	0.716(0.684–0.773)	0.713(0.641–0.794)
C index for TNM stage	0.663(0.603–0.734)	0.647(0.611–0.709)
1 year AUC for nomogram	0.701(0.612–0.751)	0.689(0.625–0.724)
3 year AUC for nomogram	0.771(0.672–0.811)	0.764(0.682–0.817)
5 year AUC for nomogram	0.762(0.673–0.821)	0.771(0.712–0.823)
1 year AUC for TNM stage	0.596(0.537–0.702)	0.643(0.605–0.683)
3 year AUC for TNM stage	0.683(0.601–0.724)	0.714(0.639–0.811)
5 year AUC for TNM stage	0.689(0.634–0.758)	0.703(0.651–0.763)

## Discussion

Colorectal adenomatous polyps are considered the main reason for the development of advanced lesions. According to current postpolypectomy surveillance guidelines, patients who have adenomas with villous elements are considered at high risk of developing advanced lesions; in addition, the size of the adenoma (> = 10 mm) would increase the risk [15]. Although colonoscopy surveillance and resection could reduce the risk of developing carcinoma, the risk of CRC after adenoma removal remains high, and the removal of adenoma does not always prevent CRC because the initial adenoma features are not well known [16, 17]. Even worse is that the knowledge of adenocarcinoma in villous adenoma is still limited to case reports and several studies. According to the current case reports, tumor recurrence was frequent due to inaccurate pathological diagnoses; however, the prognosis was good if the lesion was resected entirely [10]. Moreover, the treatment strategies for adenocarcinoma in villous adenoma differ according to different clinical behaviors [18]. Hence, it is of clinical significance to accurately predict the prognosis of patients with adenocarcinoma in villous adenoma.

In the present study, we analyzed the potential risk factors associated with colorectal adenocarcinoma in villous adenoma. In total, we determined 2831 patients who had detailed clinical information and assessed the clinical value of several characteristics by univariate and multivariate Cox regression analyses. In line with other reports [19, 20], black patients with adenocarcinoma in villous adenoma had a poor prognosis, which was caused by multiple factors, such as diet, the microbiome composition of the bowel and healthcare access [21, 22]. Similarly, age at diagnosis was an independent risk factor, which is the reason why guidelines recommend screening for CRC at 50 years old, while sex was not a prognostic factor in our study. In contrast to the findings of previous studies [19, 23], pathological grade, which is known as a prognostic factor, was not identified as an independent prognostic factor for the survival of patients with adenocarcinoma in villous adenoma. Additionally, TNM stage is known to be significantly associated with the survival of patients, and we also demonstrated that it could act as an independent predictive factor. Tumor size greater than 5 cm was considered a risk factor in our study because large tumors are not sensitive to chemotherapy and are more easily invasive [24]. Regarding the number of tumors, we found that it was an independent risk factor for OS, which is consistent with the findings of a previous report [25]. However, the number of tumors was not related to CSS, which suggests that the number of tumors mainly affects the rate of death due to other causes.

Nomograms have been successfully established to predict the survival of many tumor types and are considered a more accurate model than the 7th AJCC staging system [26–28]. To the best of our knowledge, no nomogram has been established to predict the survival of patients with adenocarcinoma in villous adenoma. Based on the results of multivariate analysis, we constructed a nomogram to evaluate the CSS of patients using the SEER database. For the nomogram predictions of 1-, 3- and 5-year CSS, age, T stage, N stage, and M stage were included in the analysis. The C-index, which was used to estimate the correlation between the predicted probability and actual event, was 0.716 (95% CI, 0.684–0.773) in the internal cohort and 0.713 (95% CI, 0.641–0.794) in the external cohort, which indicated that the nomogram was reliable. However, race and tumor size were not used to build the nomogram plot because the AIC value was too large. AIC is considered an important criterion for variable sieving and has been used in many studies [29, 30]. Moreover, according to the results of the ROC curve and DCA, the nomogram has better clinical usability than the 7th TNM staging system. Therefore, to some extent, we could evaluate the prognosis of patients by the nomogram other than TNM staging because of high reliability. According to the total score, we could determine whether patients need further chemotherapy after surgery. In that way, we could individualize the treatment of patients. In addition, we will next improve and perfect this work in a future study by collecting data for our own patients, also we will perform some experiments about adenocarcinoma in villous adenoma to investigate what differences were between adenocarcinoma in villous adenoma and conditional colorectal cancer.

Of course, our study has some limitations that should be noted. First, the TNM stage we analyzed was according to the 7th AJCC staging system, which was not the latest and may reduce the effectiveness. Then, our nomograms were constructed only by the SEER database, leading to potential selection bias. However, we developed the nomogram and verified its validity in the internal and external cohorts, which made our results more reliable. In addition, the use of AIC could make our model better by avoiding overfitting and underfitting effects. Although this nomogram performed well in the two cohorts, it should be applied with great caution when assessing the risk of 1-, 3- and 5-year survival. In the future, we will collect relevant data to incorporate the factors above into further research. Next, our manuscript has not included other characteristics, such as hematological biomarkers and molecular parameters. As some studies suggested, combining some hematological biomarkers, such as HGB, neutrophils and LDH, can promote the predictive ability of a nomogram [31], while molecular parameters, including miRNA, CpG methylation and circular RNA, have been demonstrated to be useful for predicting the survival of patients [32–34]. Therefore, we will improve and perfect this work in our future study by combining these characteristics.

## Conclusions

In this study, we found that age at diagnosis, tumor size, T stage, N stage, race and M stage were identified as risk factors for CSS in our patient sample. In addition, we constructed nomograms to predict the survival of patients and found that compared to 7th TNM staging, the nomograms could serve as a good and effective tool for survival evaluation by calculating calibration plots and ROC curves.

## Supplementary information

**Additional file 1: Supplementary Figure 1.** The flow chart of extracted patients from the SEER database.

**Additional file 2: Supplementary Figure 2.** OS curves for all patients according to different variables. (A) Age, (B) sex, (C) tumor number, (D) T stage.

**Additional file 3: Supplementary Figure 3.** OS curves for all patients according to different variables. (A) N stage, (B) M stage, (C) pathological grade type, (D) race.

**Additional file 4: Supplementary Figure 4.** OS curves for all patients according to tumor size.

**Additional file 5: Supplementary Figure 5.** Analysis of CSS for all patients according to different variables. (A) Age, (B) sex, (C) tumor number, (D) T stage.

**Additional file 6: Supplementary Figure 6.** Analysis of CSS for all patients according to different variables. (A) N stage, (B) M stage, (C) pathological grade type, (D) race.

**Additional file 7: Supplementary Figure 7.** Analysis of CSS for all patients according to M stage.

**Additional file 8: Supplementary Table 1.** the detail information about different variables according to.

## Data Availability

Not Applicable.

## Abbreviations

CRC: Colorectal cancer
SSA: Sessile serrated adenomas
VA/TVAs: Villous/tubulovillous adenomas
ESD: Endoscopic submucosal dissection
SEER: Surveillance, Epidemiology, and End Results
AIC: Akaike Information Criterion
OS: Overall survival
CSS: Cancer-specific survival
