# Supplementary figures and images for: Nomograms that predict the survival of patients with adenocarcinoma in villous adenoma of the colorectum: a SEER-based study
Source: BMC Cancer. 2020 Jun 29;20:608. doi: 10.1186/s12885-020-07099-3 (PMC7325241; doi:10.1186/s12885-020-07099-3)

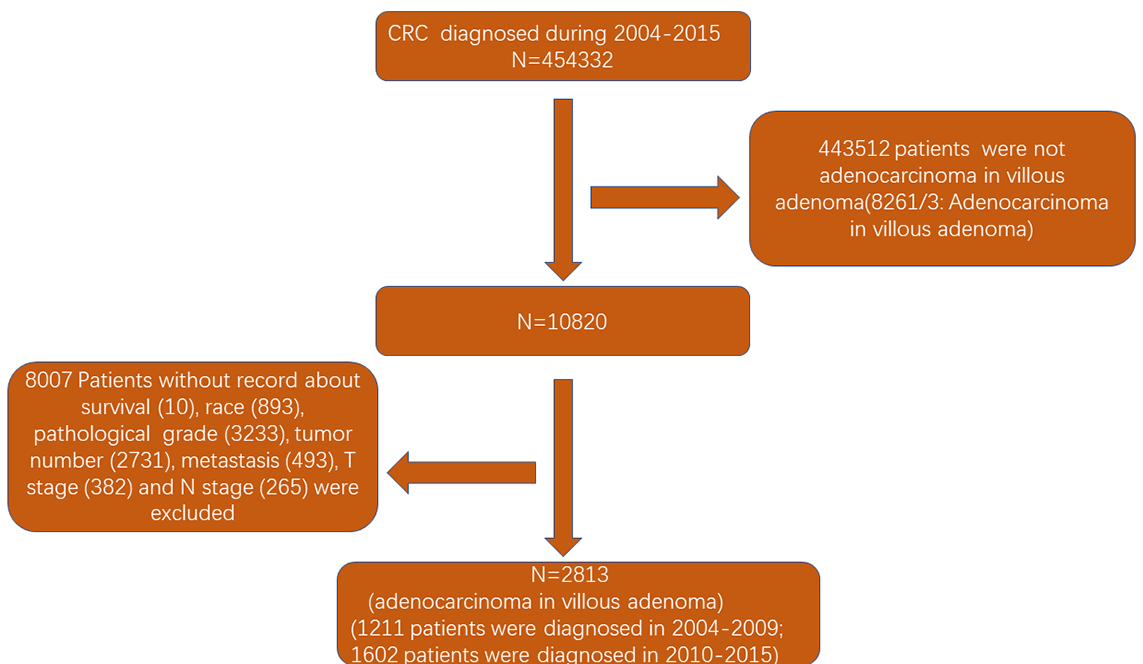

Supplement: Supplementary file 1 — Additional file 1: Supplementary Figure 1. The flow chart of extracted patients from the SEER database. [file 12885_2020_7099_MOESM1_ESM.tif]

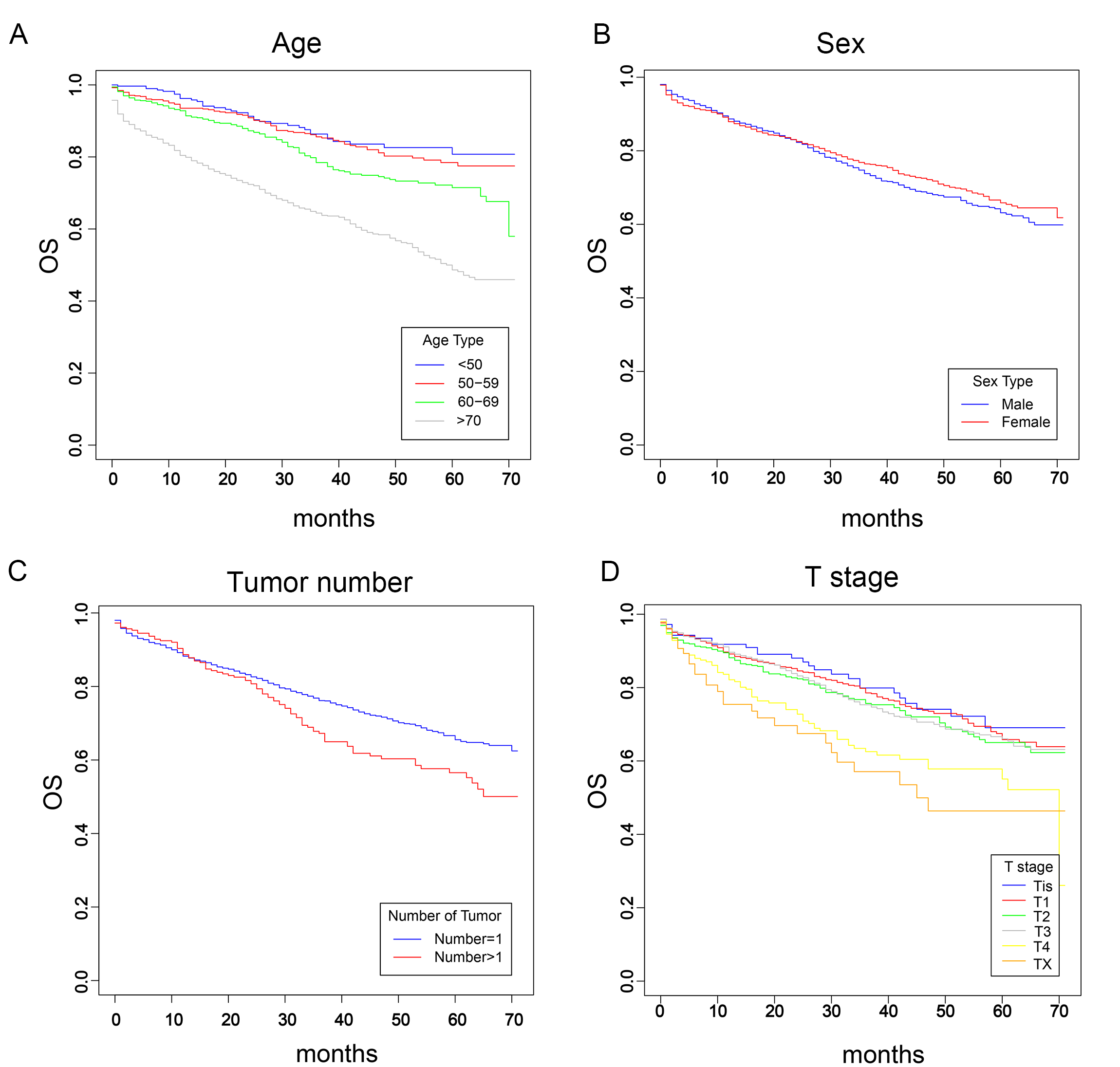

Supplement: Supplementary file 2 — Additional file 2: Supplementary Figure 2. OS curves for all patients according to different variables. (A) Age, (B) sex, (C) tumor number, (D) T stage. [file 12885_2020_7099_MOESM2_ESM.tif]

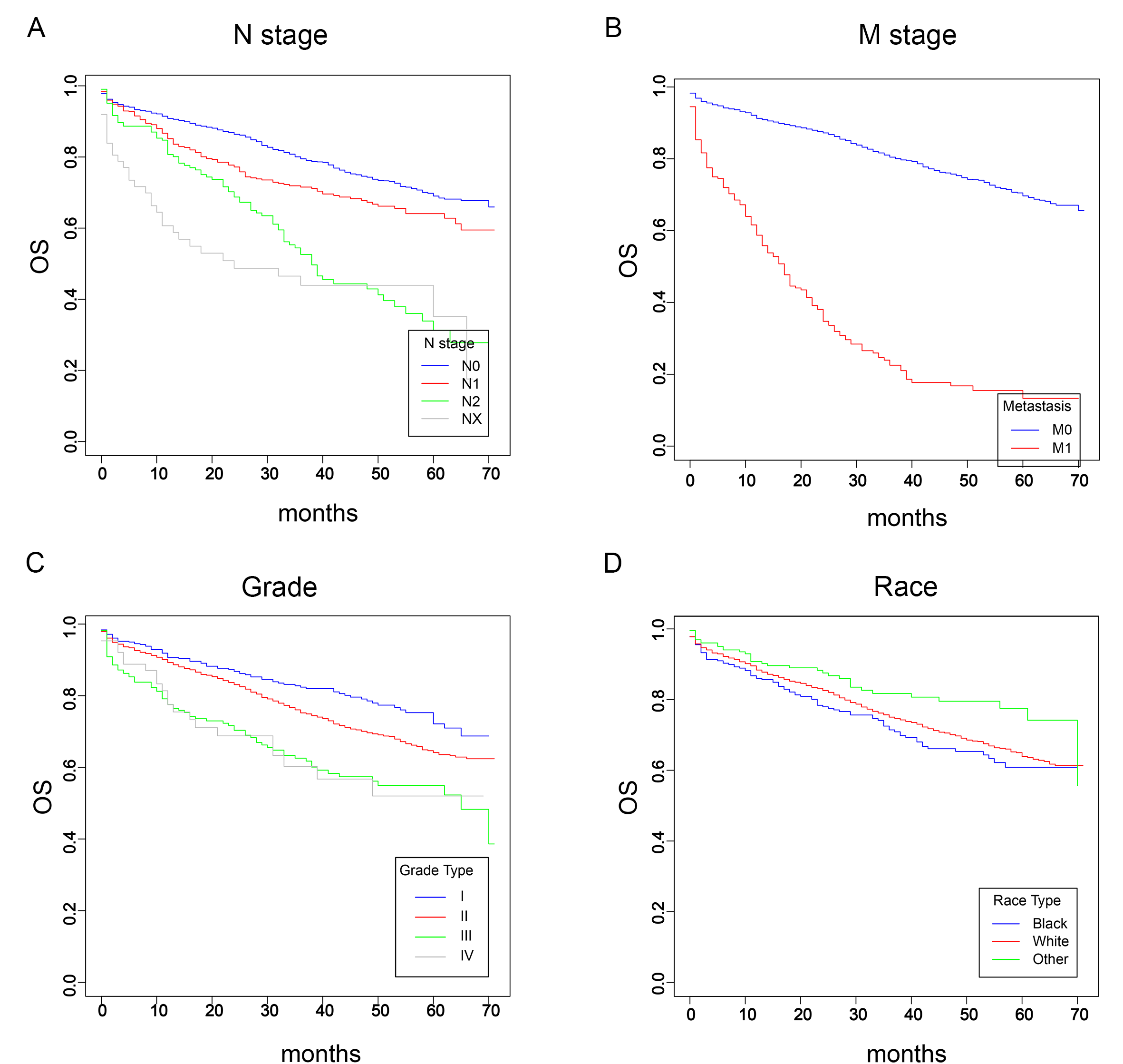

Supplement: Supplementary file 3 — Additional file 3: Supplementary Figure 3. OS curves for all patients according to different variables. (A) N stage, (B) M stage, (C) pathological grade type, (D) race. [file 12885_2020_7099_MOESM3_ESM.tif]

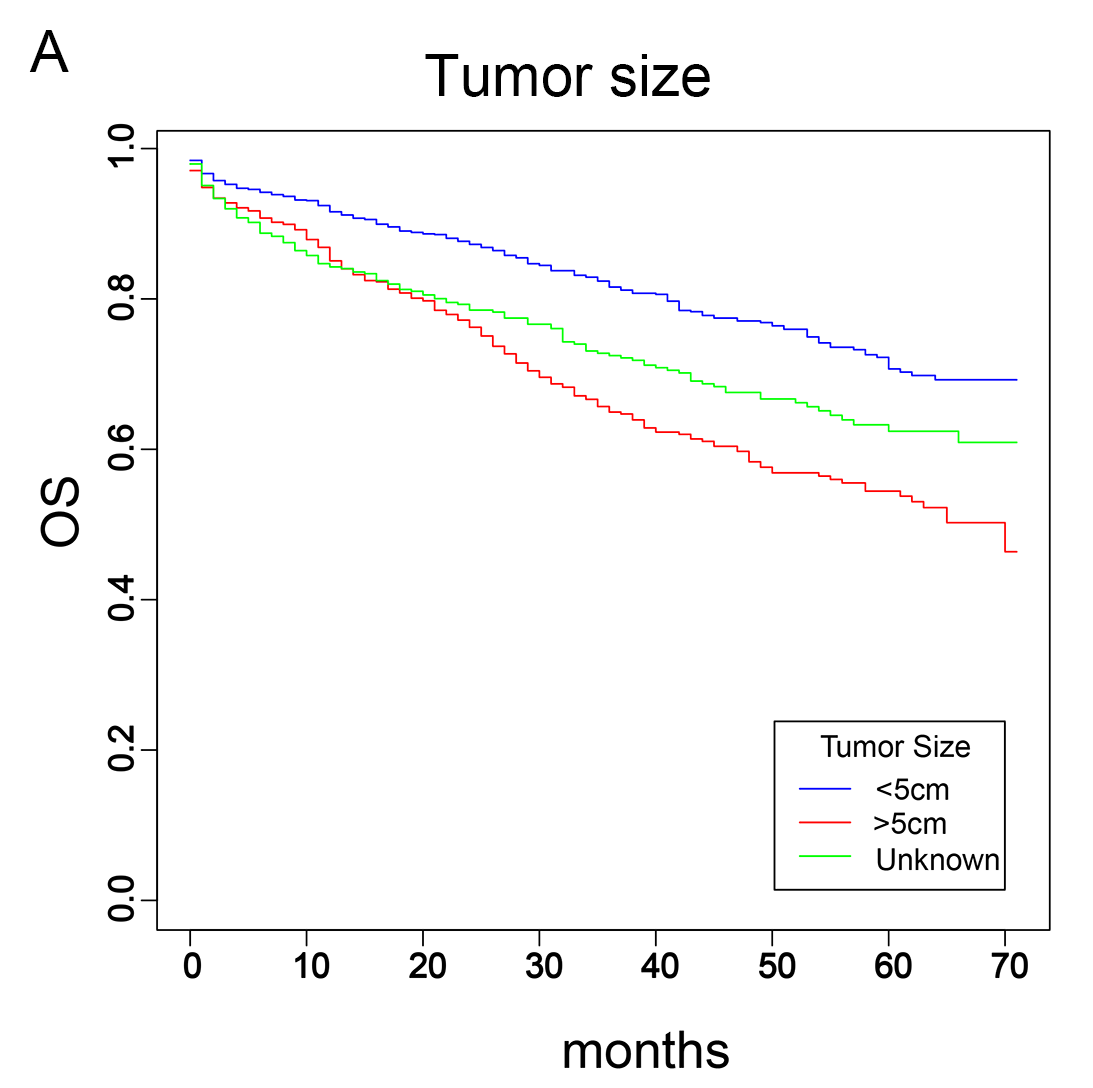

Supplement: Supplementary file 4 — Additional file 4: Supplementary Figure 4. OS curves for all patients according to tumor size. [file 12885_2020_7099_MOESM4_ESM.tif]

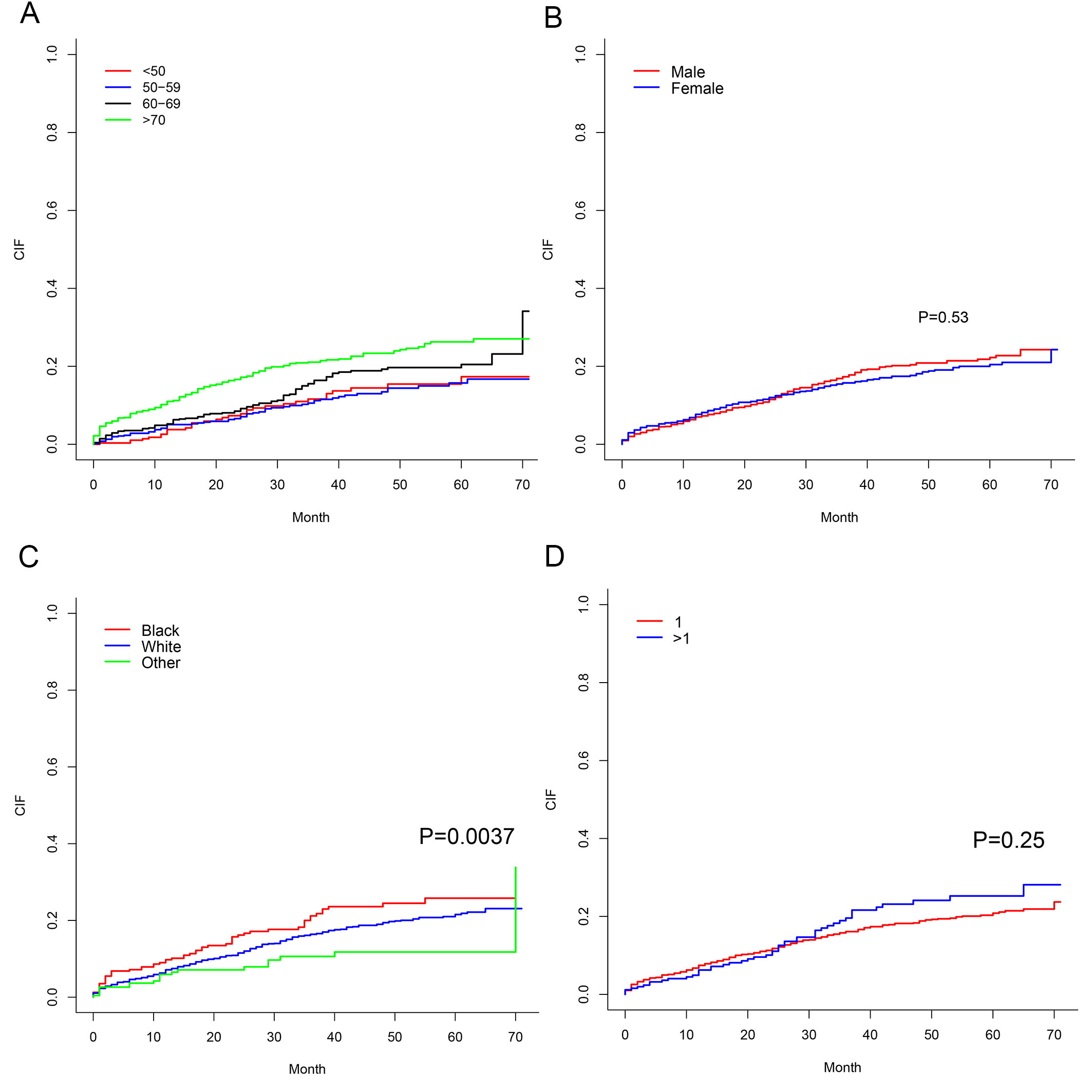

Supplement: Supplementary file 5 — Additional file 5: Supplementary Figure 5. Analysis of CSS for all patients according to different variables. (A) Age, (B) sex, (C) tumor number, (D) T stage. [file 12885_2020_7099_MOESM5_ESM.tif]

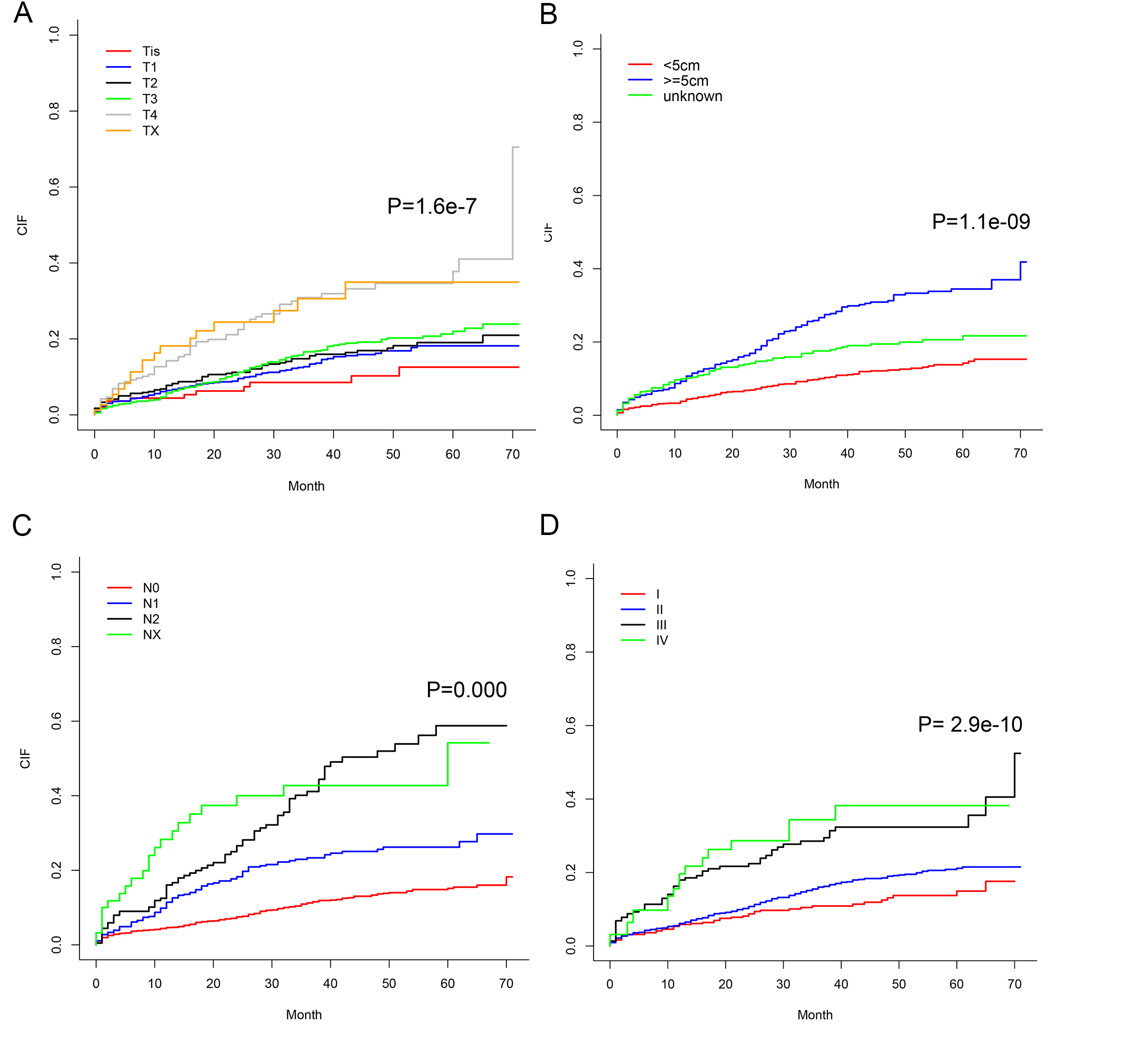

Supplement: Supplementary file 6 — Additional file 6: Supplementary Figure 6. Analysis of CSS for all patients according to different variables. (A) N stage, (B) M stage, (C) pathological grade type, (D) race. [file 12885_2020_7099_MOESM6_ESM.tif]

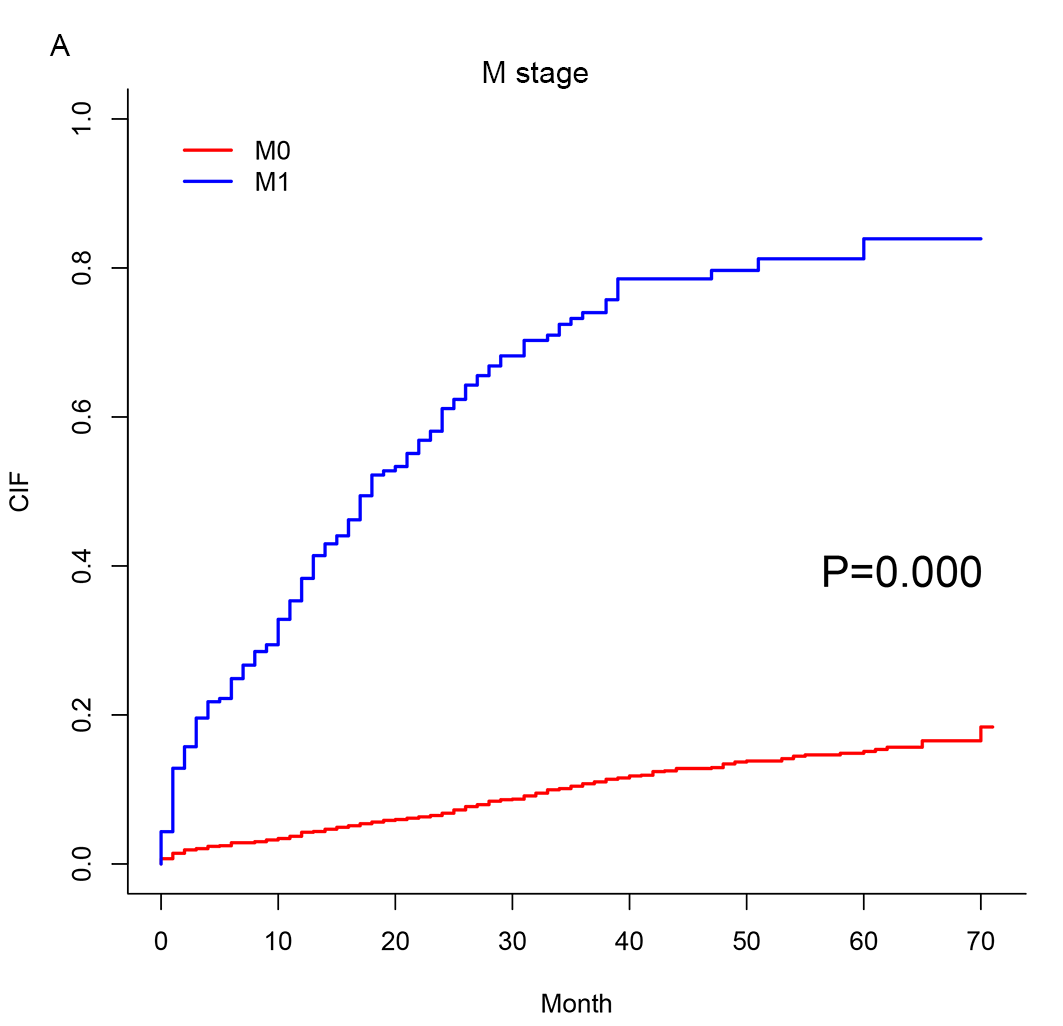

Supplement: Supplementary file 7 — Additional file 7: Supplementary Figure 7. Analysis of CSS for all patients according to M stage. [file 12885_2020_7099_MOESM7_ESM.tif]
