# Supplementary material for: Nomograms that predict the survival of patients with adenocarcinoma in villous adenoma of the colorectum: a SEER-based study
Source: BMC Cancer. 2020 Jun 29;20:608. doi: 10.1186/s12885-020-07099-3 (PMC7325241; doi:10.1186/s12885-020-07099-3)
Supplement: Supplementary file 8 — Additional file 8: Supplementary Table 1. the detail information about different variables according to. [file 12885_2020_7099_MOESM8_ESM.docx]

**Supplementary Table 1:** the detail information about different variables according to

| Variables | Total | 2004 | 2005 | 2006 | 2007 | 2008 | 2009 | 2010 | 2011 | 2012 | 2013 | 2014 | 2015 |
| --- | --- | --- | --- | --- | --- | --- | --- | --- | --- | --- | --- | --- | --- |
| Total | 8007 | 751 | 709 | 712 | 715 | 658 | 688 | 669 | 655 | 564 | 610 | 605 | 578 |
| Race | 893 | 88 | 79 | 84 | 76 | 88 | 65 | 77 | 54 | 69 | 73 | 76 | 64 |
| Pathology Grade | 3233 | 301 | 286 | 278 | 299 | 245 | 292 | 267 | 283 | 254 | 249 | 239 | 240 |
| Lymph node metastasis | 265 | 25 | 30 | 25 | 25 | 19 | 23 | 23 | 21 | 18 | 17 | 19 | 20 |
| Metastasis | 493 | 50 | 47 | 43 | 48 | 46 | 45 | 43 | 40 | 36 | 35 | 33 | 27 |
| T stage | 382 | 42 | 36 | 36 | 37 | 31 | 33 | 34 | 29 | 30 | 26 | 24 | 24 |
| Tumor number | 2731 | 245 | 231 | 246 | 230 | 229 | 230 | 225 | 218 | 220 | 210 | 214 | 203 |
